# H_2_O_2_ production rate in *Lactobacillus johnsonii* is modulated via the interplay of a heterodimeric flavin oxidoreductase with a soluble 28 Kd PAS domain containing protein

**DOI:** 10.3389/fmicb.2015.00716

**Published:** 2015-07-14

**Authors:** Ricardo B. Valladares, Christina Graves, Kaitlyn Wright, Christopher L. Gardner, Graciela L. Lorca, Claudio F. Gonzalez

**Affiliations:** ^1^Department of Microbiology and Cell Science, Genetics Institute and Institute of Food and Agricultural Sciences, University of FloridaGainesville, FL, USA; ^2^Department of Periodontology, College of Medicine, University of FloridaGainesville, FL, USA

**Keywords:** *Lactobacillus johnsonii*, hydrogen peroxide, FMN reductase, PAS domain, probiotics

## Abstract

Host and commensals crosstalk, mediated by reactive oxygen species (ROS), has triggered a growing scientific interest to understand the mechanisms governing such interaction. However, the majority of the scientific studies published do not evaluate the ROS production by commensals bacteria. In this context we recently showed that *Lactobacillus johnsonii* N6.2, a strain of probiotic value, modulates the activity of the critical enzymes 2,3-indoleamine dioxygenase *via* H_2_O_2_ production. *L. johnsonii* N6.2 by decreasing IDO activity, is able to modify the tryptophan/kynurenine ratio in the host blood with further systemic consequences. Understanding the mechanisms of H_2_O_2_ production is critical to predict the probiotic value of these strains and to optimize bacterial biomass production in industrial processes. We performed a transcriptome analysis to identify genes differentially expressed in *L. johnsonii* N6.2 cells collected from cultures grown under different aeration conditions. Herein we described the biochemical characteristics of a heterodimeric FMN reductase (FRedA/B) whose *in vitro* activity is controlled by LjPAS protein with a typical Per-Arnst-Sim (PAS) sensor domain. Interestingly, LjPAS is fused to the FMN reductase domains in other lactobacillaceae. In *L. johnsonii*, LjPAS is encoded by an independent gene which expression is repressed under anaerobic conditions (>3 fold). Purified LjPAS was able to slow down the FRedA/B initial activity rate when the holoenzyme precursors (FredA, FredB, and FMN) were mixed *in vitro.* Altogether the results obtained suggest that LjPAS module regulates the H_2_O_2_ production helping the cells to minimize oxidative stress in response to environmental conditions.

## Introduction

Studies concerning the production of ROS conventionally emphasize the negative consequences of an extreme loss of redox homeostasis. Accumulation of ROS results in damaging and irreversible modifications to proteins, nucleic acids, and lipids. The oxidative stress resulting from this redox imbalance underpins the radical theory of aging and is cited in the etiology of numerous diseases. However, it is also clear that ROS production serves an essential signaling function within and between cells within tissues. Of the candidate molecular species, the beneficial signaling properties of H_2_O_2_ are well-characterized (Veal and Day, 2011). H_2_O_2_ is ubiquitously generated during metabolism in the presence of oxygen, both as a direct product of enzymatic reactions and an indirect product of O_2_^•-^ dismutation. Through its limited oxidant activity on Fe–S centers and cysteine thiols, H_2_O_2_ serves as a vital biological messenger capable of stimulating adaptive processes in diverse cell types.

Several recent studies emphasize a growing appreciation for the significance of H_2_O_2_ in microbe-host cell crosstalk. By stimulating host cell NAD(P)H oxidase activity, bacterial members of the microbiota can indirectly increase host cell H_2_O_2_ levels (Bae et al., 2010; De Deken et al., 2014). This host cell response can positively influence host cell physiology through redox sensitive pathways, resulting in decreased inflammatory signaling, improved barrier restitution, and decreased pathogen growth (Voltan et al., 2008; Lin et al., 2009; Swanson et al., 2011; Wentworth et al., 2011). However, a majority of these studies do not evaluate the production of ROS by the commensal bacteria. Many commensal intestinal bacteria adhere closely to the intestinal mucosa, where oxygen gradients steeply rise approaching the host epithelium. In addition, many of these bacteria have been characterized to produce significant quantities of ROS *in vitro* (Marty-Teysset et al., 2000; Pridmore et al., 2008; Voltan et al., 2008). The potential role of microbe generated ROS in microbiota-intestinal epithelium crosstalk *in vivo* is noteworthy given that extracellular H_2_O_2_ can readily diffuse through cell membranes and be directly imported through aquaporins (Miller et al., 2010). However, the short-lived nature of ROS, combined with a deficit of molecular tools for their study *in vivo*, has impeded direct and thorough characterization of bacterial H_2_O_2_ production along the host intestinal mucosa.

The closely related bacterial species belonging to the acidophilus group of lactobacilli, including *L. acidophilus, L. gasseri*, and *L. johnsonii*, are members of the mammalian intestinal and vaginal microbiota. Several strains belonging to this group are also marketed as probiotics due to their ability to elicit a variety of beneficial physiological effects in their hosts (Bull et al., 2013). Our group isolated and characterized *L. johnsonii* N6.2, an intestinal commensal capable of minimizing autoimmune diabetes in the BioBreeding rat model (Lai et al., 2009; Roesch et al., 2010; Valladares et al., 2010, 2013; Kingma et al., 2011). During our analysis of the potential mechanisms underlying this protective phenotype, we found that H_2_O_2_ production by *L. johnsonii* inhibited the activity of the redox sensitive immunoregulatory protein indoleamine 2,3-dioxygenase in intestinal epithelial cells. This finding correlated with increased ileum lumen H_2_O_2_ production, decreased plasma kynurenine levels, and an increase in IL-17 producing T-helper cells in the mesenteric lymph nodes of *L. johnsonii-*fed rats (Lau et al., 2011). Overall, these findings support the significance of bacterially produced H_2_O_2_ as a molecular cue influencing host health and immunological development.

Characterizing the production of ROS by human commensal bacteria and evaluating the relevance of these signaling molecules in microbe–host interactions are active areas of investigation. As an extension of our previous work, we sought to identify proteins in *L. johnsonii* N6.2 involved in ROS production. We hypothesized that genes involved in ROS production would be differentially expressed under low and high oxygen growth. Using bioinformatic clues and RNA-seq, we identified, cloned, and purified two FMN oxidoreductases and a PAS sensor domain protein from *L. johnsonii* N6.2 relevant to ROS production. During our characterization of these purified protein targets, a report published by Hertzberger et al. (2014) showed that interruption of the homologous FMN oxidoreductase encoding genes in the commercial probiotic *L. johnsonii* NCC533 abolished the strain’s ability to produce H_2_O_2_. Given this encouraging genetic work, we continued with enzymological characterization of this novel protein complex. Here, we describe the identification, purification, and enzymological characterization of a heterodimeric FMN oxidoreductase and PAS sensor domain protein from *L. johnsonii* N6.2. Finally, we evaluate the H_2_O_2_ production by this protein complex in a tissue culture environment by comparing *L. johnsonii* wild type and *fredA/B* mutant strains.

## Materials and Methods

### Culture Growth Conditions

*Lactobacillus johnsonii* N6.2 (Valladares et al., 2010) was grown in MRS media (Remel, Lenexa, KS, USA) at 37°C under static conditions in screw cap tubes for microaerobic growth or in flasks aerated at 200 rpm for aerobic growth. For expression analysis, cultures were inoculated at 1% v/v from overnight cultures and grown in static or aerated conditions until OD_600_ = 0.5, pelleted at 8,000 × *g*, and flash frozen in liquid nitrogen. *Escherichia coli* strains were grown in standard Luria Bertani media at 37°C and 250 rpm. When indicated, antibiotics were utilized at the following concentrations: ampicillin 100 μg/ml, kanamycin 25 μg/ml, streptomycin 50 μg/ml, and tetracycline 10 μg/ml. HT-29 intestinal epithelial cells were grown in RPMI media supplemented with 10% fetal bovine serum at 37°C with 5% CO_2_. Strains and plasmids used in this work are summarized in **Table 1**.

**Table 1:** Strains and plasmids used in this study.

Strains	Description	Source
*Lactobacillus johnsonii* N6.2	Wild type strain isolated from BioBreeding Diabetes Resistant Rats	Lai et al. (2009)
*L. johnsonii* NCC533	Strain from Nestle Culture Collection	Hertzberger et al. (2014)
*L. johnsonii* NCC9359	*ΔLJ_0548 ΔLJ_0549*	Hertzberger et al. (2014)
HT-29 epithelial cells	*Homo sapiens* colorectal adenocarcinoma	ATCC
*Escherichia coli* strains		
DH5α	F– Φ80*lac*ZΔM15 Δ(*lac*ZYA-*arg*F) U169 *rec*A1 *end*A1 *hsd*R17 *pho*A *sup*E44 – *thi*-1 *gyr*A96 *rel*A1	Invitrogen
BL21-Rosetta (DE3)	F– ompT hsdSB(rB – mB –) gal dcm (DE3) pRARE	Novagen
JM109	e14– recA1 endA1 gyrA96 thi-1 hsdR17 supE44 relA1 D(lac-proAB) [F traD36 proABlacIqZDM15]	Stratagene
KDZifΔZ	[F’lacproA+,B+(lacIq lacPL8)/araD(gpt-lac)5 (DspoS3::cat)]; Km^r^	Charity et al. (2009)
Lj-FRedA	BL21-Rosetta (DE3) carrying p15TV-*fredA*; Amp^r^	This work
Lj-FRedB	BL21-Rosetta (DE3) carrying p15TV-*fredB*; Amp^r^	This work
Lj-LjPAS	BL21-Rosetta (DE3) carrying p15TV-*ljpas*; Amp^r^	This work
Coex-FRedA/LjPAS	BL21-Rosetta (DE3) carrying p15TV-*fredA* and pCDF-*ljpas*; Amp^r^, Str^r^	This work
Coex-LjPAS/FRedA	BL21-Rosetta (DE3) carrying p15TV-*ljpas* and pCDF-*fredA;* Amp^r^, Str^r^	This work
Copur-FRedA/FRedB	BL21-Rosetta (DE3) carrying p15TV-*fredA* and pCDF-his-*fredB*; Amp^r^, Str^r^	This work
RV-1	KDZifΔZ carrying pACTR-AP-Zif and pBRGP-ω; Km^r^, Amp^r^, Tet^r^	This work
RV-2	KDZifΔZ carrying pBR-*fredA* and pACTR-AP-Zif; Km^r^, Amp^r^, Tet^r^	This work
RV-3	KDZifΔZ carrying pBRGP-ω and pACTR-*fredB*; Km^r^, Amp^r^, Tet^r^	This work
RV-4	KDZifΔZ carrying pBR-*fredA* and pACTR-*fredB*; Km^r^, Amp^r^, Tet^r^	This work

**Plasmids**	**Description**	**Source**

p15TV-L	Expression vector for protein purification, GenBank accession: EF456736; Amp^r^	SGC, Toronto
p15TV-*fredA*	p15TV-L carrying gene encoding FRedA from *L. johnsonii* N6.2	This work
p15TV-*fredB*	p15TV-L carrying gene encoding FRedB from *L. johnsonii* N6.2	This work
p15TV-*ljpas*	p15TV-L carrying gene encoding LjPAS from *L. johnsonii* N6.2	This work
pCDF-1b	Expression vector for protein expression; Str^r^	Novagen
pCDF-*ljpas*	pCDF-1b carrying gene encoding LjPAS in NcoI and NotI (excludes His_6_ tag)	This work
pCDF-*fredA*	pCDF-1b carrying gene encoding FRedA in NcoI and NotI (excludes His_6_ tag)	This work
pCDF-his-*fredB*	pCDF-1b carrying gene encoding FRedB in BamHI and NotI (N-terminal His_6_ tag)	This work
pACTR-AP-Zif	Plasmid for N-terminal Zif protein fusion; Tet^r^	Charity et al. (2009)
pBRGP-ω	Plasmid for N-terminal fusions to ω subunit of *E. coli* RNA polymerase; Car^r^, Amp^r^	Charity et al. (2009)
pACTR-*fredB*	pACTR-AP-Zif carrying a gene encoding FRedB from *L. johnsonii* N6.2	This work
pBR-*fredA*	pBRGP-ω carrying a gene from *L. johnsonii* N6.2	This work

### RNA-Seq and Quantitative Real Time PCR Analysis of Static and Aerated *L. johnsonii* N6.2

RNA extractions were performed as previously described (Moreno et al., 2001). Briefly, cell lysis was achieved using equal volumes of 0.1 mm zirconia beads:extraction buffer with 30 s rounds of vortexing and cooling on ice over 5 min. The acidified lysate was extracted twice using a 25:24:1 acid phenol:chloroform:isoamyl alcohol solution. The final RNA product was precipitated from the aqueous phase with 100% isopropanol, rinsed with 95% EtOH, and resuspended in nuclease-free H_2_O. Samples were treated with DNase I before RNA integrity was determined using an Agilent 2100 Bioanalyzer. After quality check, ribosomal RNA was depleted using the Ribo-Zero bacterial rRNA removal kit (Epicentre, Madison, WI, USA). Single end, directional libraries were created using the ScriptSeq RNA-Seq Library Preparation kit (Epicentre), followed by sequencing using the Illumina Hiseq2000 Sequencer.

Sequencing data were analyzed using a pipeline within the Galaxy bioinformatics suite (http://galaxyproject.org/; Goecks et al., 2010). Briefly, raw data Sanger formatting was performed using the FastQ Groomer Tool followed by FastQC processing to verify read quality prior to downstream analysis (Cock et al., 2010). Sequence data were quality filtered using the windowed adaptive trimming tool Sickle and mapped to the recently published *L. johnsonii* N6.2 reference genome using eXpress (Leonard et al., 2014). Reads were normalized using the reads per kb of transcript per million reads mapped (RPKM) approach (Mortazavi et al., 2008), and transcript abundance was compared between aerated and static *L. johnsonii* N6.2 cultures.

For quantitative real time PCR, RNA was extracted from static and aerated *L. johnsonii* cultures as described above. Single stranded cDNA was synthesized using iScript cDNA synthesis kit (BioRad, Hercules, CA, USA). Reactions were performed in duplicate with an iCycler detection system (BioRad) using iQ SYBR Green Supermix and 200 nM of each primer in a reaction volume of 25 μL. Expression between aerated and static cultures were compared using iCycler Software and the C_t_ (2^-ΔΔ^*^C^*_T_) method of relative transcript analysis with the RNA polymerase sigma factor gene *rpoD* serving as an internal control. Primer sequences are listed in **Table 2**.

**Table 2:** Oligonucleotides used in this study.

Protein purification	Sequence 5′–3′
P15TV-L FRedA-F	*tgtatttccagggc*atgaaattactagcaattgttgg
P15TV-L FRedA-R	*caagcttcgtcatca*ttatttttgagcttgttcaacacaaa
P15TV-L FRedB-F	*tgtatttccagggc*atgaaactctttgccattgt
P15TV-L FRedB-R	*caagcttcgtcatca*ctacttacttacttcagcataa
P15TV-L LjPAS-F	*ttgtatttccagggc*atggcagaacctaaatggtta
P15TV-L LjPAS-R	*caagcttcgtcatca*ttaatactctgaagcaccaga
FRedA pCDF BamHI-Fw	cgcggatcccatgaaattactagcaattgttg
FRedA pCDF NotIRv	atatcaatatgcggccgcattatttttgagcttgttcaaca
FRedB pCDF BamHI-Fw	cgcggatcccatgaaactctttgccattgta
FRedB pCDF NotIRv	atatcaatatgcggccgcactacttacttacttcagcat

**Coexpression**	

FRedB pCDF NcoI-Fw	gacatgccatggaactctttgccattgt
FRedB pCDF NotI-Rv	atatcaatatgcggccgcactacttacttacttcagcat
FRedA pCDF NcoI-Fw	gacatgccatggaattactagcaattgttg
FRedA pCDF NotI-Rv	tatatgcggccgctttttgagcttgttcaacaaat
LjPAS pCDF NcoI-Fw	gacatgccatggtggcagaacctaaatggtt
LjPAS pCDF NotI-Rv	tatatgcggccgcatactctgaagcaccagaa

**Dual hybrid**	

FRedA NdeI-Fw	ggaattccatatgatgaaattactagcaattgttg
FRedA NotI Rv	tatatgcggccgctttttgagcttgttcaacaaat
FRedB NdeI-Fw	ggaattccatatgatgaaactctttgccattgta
FRedB NotI Rv	tatatgcggccgccttacttacttcagcataaaaa

**qRT-PCR**	

Alcohol/acetylaldehyde dehydrogenase-F	gtggtcatactgcgggagtt
Alcohol/acetylaldehyde dehydrogenase-R	acgacatgcatccattttca
Cytochrome d oxidase 1-F	cactggtgagctctggttca
Cytochrome d oxidase 1-R	actccgccaagagttgagaa
Cytochrome d oxidase 2-F	tcgctactgatccagcacac
Cytochrome d oxidase 2-R	agctaaagcgattggcaaaa
Fructose/mannose inducible IIC-F	ttggtgcacaaggtgtcact
Fructose/mannose inducible IIC-R	tggaagcatagcaccaacag
PAS 10 containing protein (LjPAS)-F	atacaatgggcgctgttcat
PAS 10 containing protein (LjPAS)-R	acgggcattttcacttcatc
Lactate dehydrogenase-F	ttcaagctgctcgcgtacta
Lactate dehydrogenase-R	gtaagtcgcgcttagccatc
Surface protein Rib-F	aacacagccaattcacacca
Surface protein Rib-R	gctggcatttcatccttgtt
FtsZ cell division protein-F	agctgctgaagagagccaac
FtsZ cell division protein-R	tgcaattacaggagcagcac
Glutamine synthetase type I-F	tcggcgtgatattgttgaaa
Glutamine synthetase type I-R	cacctacttcgtggtgagca
Mannose/fructose/sorbose family IIC-F	tttacctggggattttcacg
Mannose/fructose/sorbose family IIC-R	aacccatcagtcaaccaagc
MocA family oxidoreductases-F	gcgagcaaaacagcctaaac
MocA family oxidoreductases-R	cttcatgggcacttgcagta
Hypothetical adhesin-F	taattctggtgcagctggtg

**qRT-PCR**	**Sequence 5′–3′**

Hypothetical adhesin-R	agcggctgcatcgttaatac
Iron–sulfur cluster protein SufB-F	gctcgcggtactatgctttc
Iron–sulfur cluster protein SufB-R	tccagctccagaatcttggt
Manganese transporter MntH-F	agaagccctggtatcctgct
Manganese transporter MntH-R	gagatggcaatgcagacaaa
Sigma factor gene *rpoD*-F	ctggagttggttctcttccca
Sigma factor gene *rpoD*-R	taacatgggtttgatgaaggct

*Underlines indicate restriction sites.*

*Italics indicate infusion recombination sequences.*

### Protein Purification

Chromosomal DNA isolation, PCR, restriction digestions, gel electrophoresis, ligations, and transformations were all performed using standard techniques (Swanson et al., 2011). Genomic DNA was isolated using DNeasy kits, plasmids were purified using QIAPrep Spin kits, and PCR products were cleaned using QIAquick kits (Qiagen, Valencia, CA, USA). All strains and primers are described in **Tables 1 and 2** respectively.

To express individual N-terminal His_6_ tagged proteins, genes encoding LjPAS, FRedA, and FRedB were PCR amplified and cloned into p15TV-L as previously described (Lorca et al., 2007). For coexpression of His_6_-LjPAS or His_6_-FRedA with untagged FRedB, FRedB was cloned into pCDF-1b using NcoI and NotI restriction sites. For copurification of His_6_-FRedB with His_6_-FRedA, FRedB was cloned into pCDF1b using BamHI and NotI restriction sites. Plasmids were sequenced for confirmation of sequence fidelity and frame prior to purification.

Recombinant plasmids were transformed individually or cotransformed into chemically competent *E. coli* BL21 (DE3; Stratagene, Santa Clara, CA, USA). Cells were cultured to OD_600_ = 0.5 and induced with 500 μM IPTG at 17°C for 16 h. After induction, cells were collected by centrifugation, resuspended in binding buffer (5 mM imidazole, 500 mM NaCl, 20 mM Tris-HCl pH 7.9) and lysed by French press. The His_6_ tagged proteins were purified from clarified lysate using nickel affinity chromatography as described previously (Pagliai et al., 2010). Purified proteins were dialyzed in 50 mM Tris-HCl buffer pH 8.00, 500 mM NaCl, and 1 mM DTT at 4°C and stored at -80°C. Standard methods for 15% SDS-PAGE were used to separate and visualize purified proteins.

### Steady-State Kinetics and Enzymatic Assays

The velocity of NADH oxidation at 37°C was followed spectrophotometrically at OD_340_ (ε = 6.22 mM^-1^ cm^-1^) over 10 min with a total reaction volume of 200 μL containing 500 ng/mL of FRedA/B in 100 mM MES buffer pH 5.5. Variable concentrations of NADH or FMN/FAD were used with a fixed concentration of the electron acceptors or electron donor, respectively. Kinetic parameters were determined by non-linear regression using Origin 8.0 (OriginLab, Northampton, MA, USA). All other enzymatic assays were performed in a total volume of 200 μL containing 500 ng/mL of FRedA and FRedB in 100 mM MES buffer pH 5.5 with 40 μM FMN. Reactions were initiated by the addition of 400 μM NADH.

### Size Exclusion Chromatography

FPLC analysis was carried out using a Tricorn Superdex 75 10/300 GL gel filtration column (GE Healthcare) connected to a Pharmacia LCC-501 Plus controller. The column was pre-equilibrated with 50 mM sodium phosphate, pH 6. Protein samples (450–500 μg) were prepared in 100 mM MES pH 6 with 150 mM NaCl. Samples were incubated 5 min at 25°C in the presence or absence of 50 μM FMN. Eluted proteins were detected by monitoring absorbance at 280 nm with a UV-M II monitor (Pharmacia). The molecular weight of samples was estimated using a combination of protein molecular weight standards, including immunoglobulin IgG (150 kDa), BSA (66 kDa), albumin (45 kDa), trypsinogen (24 kDa), cytochrome C (12.4 kDa), and vitamin B12 (1.36 kDa).

### Isothermal Titration Calorimetry

Isothermal Titration Calorimetry measurements were performed on a VP-ITC Microcalorimeter (MicroCal, Northampton, MA, USA) at 25°C. The protein was thoroughly dialyzed against 10 mM MES pH 6.0 and 150 mM NaCl. A solution of 1 mM FMN was prepared in dialysis buffer. Each titration involved a series of 5 μl injections of FMN into the protein solution. The mean enthalpies measured from injection of the ligand into the buffer were subtracted from raw titration data before analysis with Origin software (MicroCal). Titration curves were fitted by a non-linear least squares method to a function for the binding of a ligand to a macromolecule (Wiseman et al., 1989). From the curve thus fitted, the parameters ΔH (reaction enthalpy), K_A_ (binding constant, K_A_ = 1/K_D_), and *n* (reaction stoichiometry) were determined. From the values of K_A_ and ΔH, the change in free energy (ΔG) and entropy (ΔS) were calculated with the equation: ΔG = 2RTlnK_A_ = ΔH-TΔS, where R is the universal molar gas constant (8.314 J K^-1^ mol^-1^) and T is the absolute temperature.

### Hydrogen Peroxide Quantification

Hydrogen peroxide was quantified using AAP as previously described (Marty-Teysset et al., 2000). Samples were read in 96-well plates at 505 nm using a Synergy HT spectrophotometer (Biotek, Winooski, VT, USA) at 37°C. Concentrations were estimated based on standard curves run in parallel with samples. ROS were visualized in coculture experiments using the fluorescence ROS dye CellRox Deep Red reagent (Life Technologies) per the manufacturer’s instructions. Cell nuclei were visualized using DAPI and photographed using a Leica TCS SP5 confocal microscope equipped with LAS AF software (Leica Microsystems, Buffalo Grove, IL, USA).

### Bacterial Dual Hybrid Assay

Plasmid pBRGP-ω, directing the synthesis of the Gal11P-v fusion, and plasmid pACTR-AP-Zif, directing the synthesis of the zinc finger DNA-binding domain of the murine Zif268 protein fusion, have been described previously (Vallet-Gely et al., 2005; Charity et al., 2009). For the bacterial two-hybrid system, the *L. johnsonii* gene encoding FRedA was cloned into the NdeI and NotI sites of plasmid pBRGP-ω to create a RNAP-ω subunit protein fusion. The *L. johnsonii* gene encoding FRedB was cloned into the NdeI and NotI sites of plasmid pACTR-AP-Zif to create the zinc finger fusion protein pACTR-FRedB.

Plasmids were individually transformed into chemically competent *E. coli* JM109 for propagation and sequencing, followed by cotransformation into the reporter strain *E. coli* KDZifΔZ. Reporter cells were grown in LB broth at 250 rpm and 37°C. Expression of the fusion proteins was induced by the addition of 10 μM IPTG when cells reached OD_600_ = 0.2. After 2 h, cells were lysed by addition of 0.15% SDS and 1.5% chloroform in Z-buffer (60 mM Na_2_HPO_4_ 7H_2_O, 40 mM NaH_2_PO_4_ H_2_O, 10 mM KCl, 1 mM MgSO_4_, 50 mM β-mercaptoethanol) and assayed for β-galactosidase activity by following the hydrolysis of CRPG substrate by continuous reading at 570 nm. β-galactosidase activity is expressed in AUs (Miller, 1972), and background activity was determined using empty control plasmids (pACTR-AP-Zif and pBRGP-ω). For anaerobic β-galactosidase assays, cultures were grown in a sealed anaerobic container system with a CO_2_-generating GasPack (BD Biosciences).

### High Performance Liquid Chromatography

Reverse-phase HPLC was used to identify and quantify the flavin cofactor bound to purified FRedA/B. Proteins were denatured by boiling the samples for 10 min. Denatured protein was removed by centrifugation and released cofactor was analyzed using an LaChrom Elite HPLC system (Hitachi, Dallas, TX, USA) equipped with an Ascentis Express 15 cm × 4.6 mm, 3 μm RP-Amide column (Sigma-Aldrich, St. Louis, MO, USA) at 25°C. Samples were separated using an isocratic mobile phase of 20 mM NaH_2_PO_4_ pH 4, 2.5% acetonitrile, and flavin was detected by absorbance at 204 nm. Chromatograms were analyzed in EZChrom Elite 3.3.2 software (Agilent, Santa Clara, CA, USA).

### H_2_O_2_ Production in HT-29 Tissue Culture

HT-29 cells were culture at 37°C using McCoy’s 5a culture media, supplemented with 10% FBS in a 5% CO_2_ atmosphere on poly-L-lysine coated glass coverslips until the culture reaches 90% cell confluence. *Lactobacillus* strains were cultured in parallel on MRS liquid media until OD_600_ = 0.2 (∼10^7^ cell ml^-1^). Bacteria cells were washed and suspended to the appropriated cells concentration on McCoy’s 5a culture media, supplemented with 10% FBS immediately before mixing them with the HT-29 cells. The mixture (bacteria plus HT-29 cells) was incubated during 120 min in the same conditions used before for HT-29 cells culture. The cells were stained during 30 min with a solution containing McCoy’s 5a in 25 mM HEPES buffer amended with 5 μM CellRox Deep Red and nuclear Stain. After washing the excess of staining solution with PBS, the cells were fixed for imaging in 4% PFA, 10 min at 37°C. The coverslips were mounted in a glass slide with Vectashield^©^ and analyzed with confocal microscopy.

### Biostatistical Analyses

All enzymatic assays were performed in triplicate with values reported as means with standard deviations. The NADH oxidase activity of experimental groups and the β-galactosidase activity of dual hybrid constructs were compared by ANOVA with a *post hoc* Tukey HSD using GraphPad Prism version 6.0 (GraphPad, La Jolla, CA, USA). *p*-values < 0.05 were considered significant.

## Results

### *L. johnsonii* N6.2 Transcriptome Analysis during Oxygen Stress

*Lactobacillus johnsonii* and related human commensal lactobacilli associate closely with host epithelial surfaces in the oral, intestinal, and urogenital environments, where oxygen gradients vary and steeply rise approaching the highly irrigated mucosa. To understand the physiological response of the probiotic *L. johnsonii* to the presence of oxygen, and to identify genes involved in ROS production, we characterized the *L. johnsonii* global transcription-level response to aeration using RNA-Seq (Supplementary Table S1). We hypothesized that genes involved in ROS production would be differentially expressed under low and high oxygen growth. When compared to static cells, aerated *L. johnsonii* N6.2 cells showed a significant change (>3 fold) in 43 transcripts. Twenty-three unique transcripts showed a significant increase during aeration. These included several transcripts involved in import and metabolism of antioxidant molecules such as cystine (+5.62 fold) and manganese (+13.54 fold). Transcripts corresponding to several putative cell wall-anchored mucin binding proteins (+4.40 and 4.95 fold) also increased during aeration, as did five loci belonging to the putative iron–sulfur cluster SUF operon (all > +7.13 fold). The 20 transcripts with decreased expression during aeration included acetaldehyde/alcohol dehydrogenase (-12.00 fold), all four genes in the putative cytochrome bd oxidase operon (*cydABCD*; all > -3.20 fold), several transcripts for genes involved in PTS sugar transport (all > -3.46 fold), and a fumarate reductase flavoprotein precursor (-3.07 fold). The RNA-seq results were validated by qRT-PCR using the genes encoding transcripts that showed significant changes during aeration (Supplementary Table S2).

A gene by gene analysis of the differentially expressed genes in *L. johnsonii* during low and high oxygen growth showed that a majority of the corresponding target pathways and proteins have been extensively characterized as general oxidative stress responses in other bacterial species (Puri-Taneja et al., 2007; Anjem et al., 2009; Culotta and Daly, 2013; Kang et al., 2013). In this group we also pinpointed a gene encoding a poorly characterized putative fumarate reductase flavoprotein (locus T285_08005). The transcription of this gene displayed a substantial (-3.07 fold) drop during high oxygen growth (Supplementary Table S1). This gene was systematically annotated as putative fumarate reductase. However, there is not experimental evidence supporting this biological function. A further analysis of the protein sequence showed that the protein encoded by locus T285_08005 was miss-annotated. Consequently, we further investigated the role the gene product in ROS production by *L. johnsonii* N6.2.

### Analysis of the Gene T285_08005 and Identification of a Novel System Involved in H_2_O_2_ Production

**FIGURE 1:**
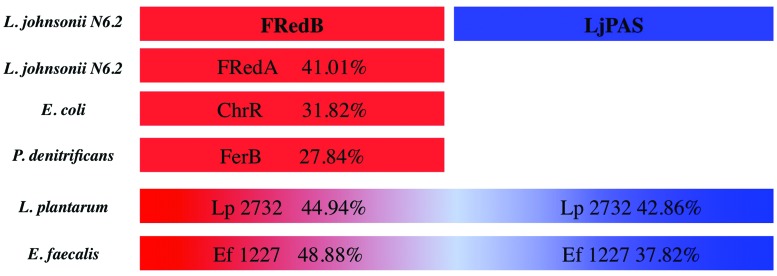
**Schematic representation of homology of *Lactobacillus johnsonii* N6.2 FRedA, FRedB, and LjPAS proteins with homologs and fusion partners in other species.** Fusion partners were identified using STRING database. Sequence homology was determined using Clustal-ω compared to *L. johnsonii* N6.2 FRedB (Red) or LjPAS (Blue) proteins. FRedB GI:560152837, FRedA GI:560152836, LjPAS GI:560152752, ChrR GI:388603952 PDB:3SVL, FerB GI:409973692 PDB:3U7R, Lp2732 GI:342242611, EF1227 GI: GI:29343261.

The encoded protein belongs to a conserved family of uncharacterized proteins in the phylum Firmicutes containing a singular PAS signaling domain. Henceforth we refer to the PAS-domain containing protein in *L. johnsonii* N6.2 as LjPAS. Analysis of LjPAS in search of predicted physical or functional protein interactions showed that other species of lactic acid bacteria also encode LjPAS homologs. Interestingly, in several close related species, this protein is fused to a putative NAD(P)H-dependent FMN reductases (**Figure 1**). This evidence redirected our research toward the fused domains categorized as NAD(P)H oxidases, including FMN reductases, because they recognized capability of producing ROS (Meehan and Malamy, 2012). Using the FMN reductase portion of the PAS-10 fusion protein in *L. plantarum*, we performed a BLASTp search in *L. johnsonii* N6.2. This search identified two sequentially encoded, putative FMN reductases with 42% identity to the *L. plantarum* fusion partner (YP_008845705 and YP_008845706). Henceforth we refer to the two consecutively encoded, putative FMN reductases in *L. johnsonii* N6.2 as FRedA and FRedB, respectively. In light of the significant change in LjPAS expression during aeration (Supplementary Tables S1 and S2), the fusion of LjPAS homologs to putative FMN reductases in other Firmicutes (**Figure 1**), and the previously described ROS producing characteristic of FMN reductases (Becker et al., 2011), we focused our subsequent work on the purification and characterization of these three proteins from *L. johnsonii* N6.2.

### Purification of FRedA, FRedB, and LjPAS Proteins of *L. johnsonii* N6.2

**FIGURE 2:**
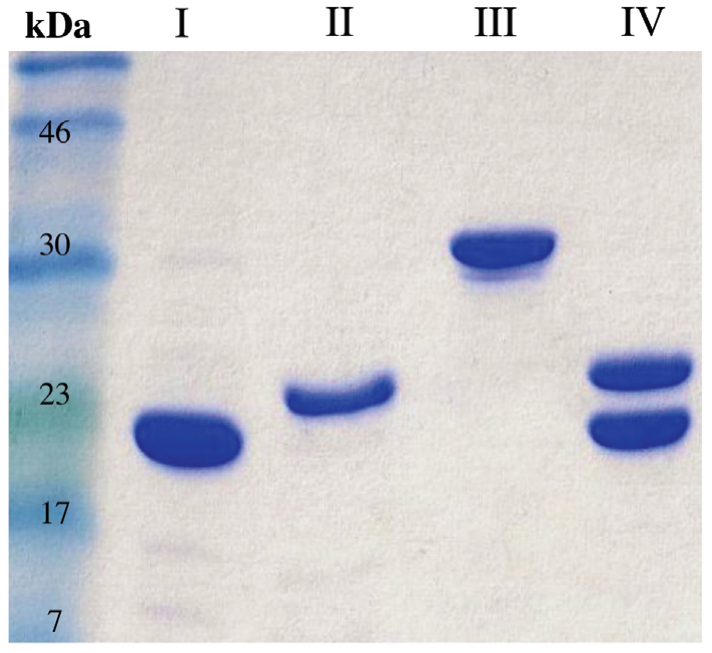
**Purification FRedA, FRedB, LjPAS, and FRedA/B.** Genes of interest were cloned with N-terminal His_6_ tags and purified from *Escherichia coli* BL21 (DE3) using nickel-affinity chromatography. (I) His_6_-FRedB (II) His_6_-FRedA + LjPAS (III) His_6_-LjPAS + FRedA (IV) Co-purified His_6_-FRedA + His_6_-FRedB.

To understand the contribution of FRedA, FRedB, and LjPAS to ROS production in *L. johnsonii* N6.2, the encoding genes were cloned and the proteins purified. In the first attempt, all the proteins were expressed one at the time using individual clones in *E. coli* BL21 strain. On these conditions only FRedB could be purified (12 mg l^-1^ culture; **Figure 2**, I). Notably, FRedB did not purify with the characteristic yellow color indicative of the binding of a flavin cofactor to FMN oxidoreductases. FRedA and LjPAS formed inclusion bodies after induction hindering the recovery of soluble purified proteins.

Co-expression of His_6_-FRedA with untagged LjPAS facilitated the synthesis of soluble His_6_-FRedA aiding high yield purification (5 mg l^-1^ culture; **Figure 2**, II). Similarly, the co-expression of His_6_-LjPAS with untagged FRedA significantly improved the recovery of soluble LjPAS by affinity chromatography (11 mg l^-1^ culture **Figure 2**, III). Interestingly, purified His_6_-FRedA also lacked the typical yellow color indicating flavin cofactor binding.

FRedA and FRedB share 41% homology and appear to be expressed in a single transcript with a 12 base pair intergenic region. Given these observations, and the finding that separately purified FRedA and FRedB lack flavin cofactor, we tested if the co-expression and purification of His_6_-FredA with His_6_-FredB would result in the purification of the active holoenzyme. The co-expression of His_6_-FRedB improved the recovery yield of His_6_-FRedA (**Figure 2**, IV). The co-purified FredA/B proteins, on these conditions, eluted with a bright yellow color indicative of flavin cofactor attachment. The presence of flavin was confirmed by HPLC analysis of the heat-denatured protein supernatant.

### FRedA/B Form a Stable Heterodimer with Two Flavin Binding Sites

**FIGURE 3:**
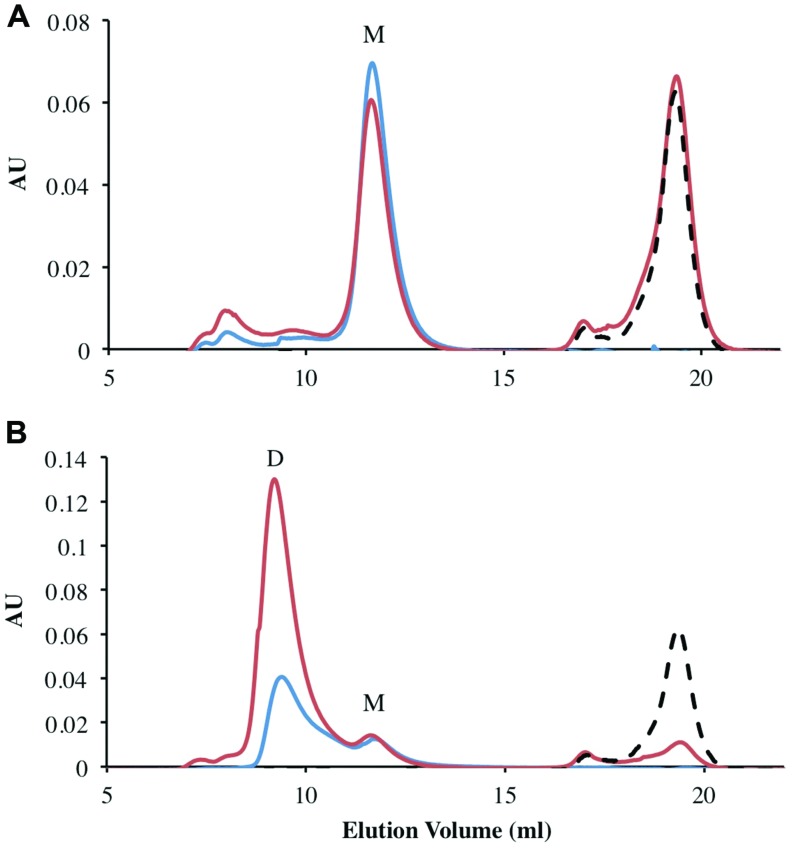
**Analysis of oligomeric status of FRedA, FRedB, and FRedA/B using size exclusion chromatography.** Proteins (250 μg total) were run alone (Blue) or after incubation with 50 μM FMN (Red). The elution profile of the 50 μM FMN control is indicated by the dotted line (Black). Peaks corresponding to the expected elution volume for monomeric (∼21 kDa) and dimeric (∼43 kDa) proteins are indicated by M and D, respectively. **(A)** The elution profile for individually purified FRedB; **(B)** the elution profile for copurified FRedA/B.

All previously characterized FMN oxidoreductases are reported to form homodimers and homotetramers in solution (Eswaramoorthy et al., 2012; Sedláček et al., 2014). To determine the oligomeric state of the individually purified FRedA, FRedB, and copurified proteins (FRedA/B), size exclusion chromatography was performed. The elution profile of FRedB displayed a single peak with an apparent molecular weight of 22 kDa, corresponding to a monomer (**Figure 3A**). A similar elution profile was obtained after incubation of FRedB with 50 μM FMN (**Figure 3A**). FRedA, on the contrary, underwent aggregation during size exclusion analysis and consistently eluted in the void volume. Incubation of either protein with FMN had no effect on the elution profile obtained.

The elution profile of the copurified FRedA/B complex displayed a significant peak at a molecular weight of 43 kDa, corresponding to a heterodimer (**Figure 3B**). A smaller peak corresponding to the 21 kDa monomer also eluted. Interestingly, a significant increase in the absorbance of the 43 kDa peak was observed after incubation of the FRedA/B complex with 50 μM (**Figure 3B**). This result supports the binding of FMN to the heterodimer complex, and suggests that both the FRedA and FRedB monomers are required for the formation of the FMN binding pocket.

**FIGURE 4:**
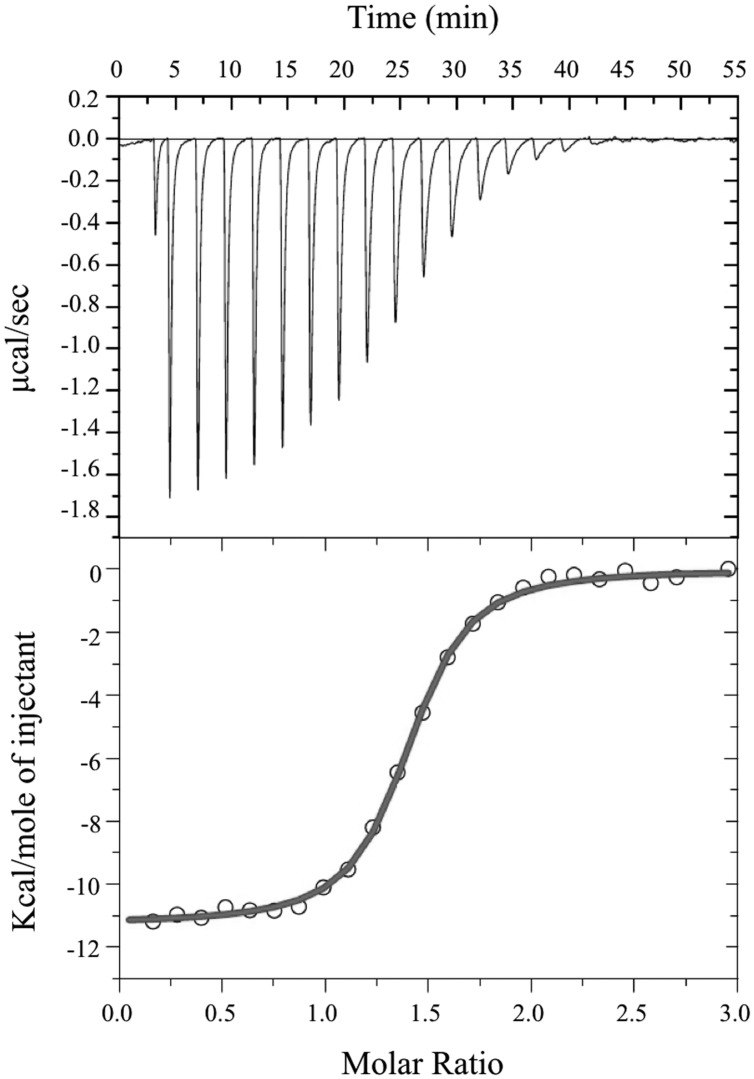
**Isothermal titration calorimetric analysis of FMN binding to FRedA/B.** For ITC, measurement of heat changes **(upper)** and integrated peak areas **(lower)** of a series of 5 μl injections of 1 mM FMN into a 40 μM protein solution prepared in 10 mM Tris pH 6.0, 150 mM NaCl are shown. Experiments were carried out at 25°C.

Size exclusion chromatography experiments suggested that the pockets of the purified protein were not completely saturated with FMN cofactor. The theoretical binding capacity for the FRedA/B complex is 2 moles of FMN per 1 mole of protein, since both FRedA and FRedB each contain one predicted FMN binding site. The quantification of FMN bound to purified FRedA/B complex revealed a stoichiometric relationship of 0.58 moles FMN to 1 mole of dimer complex. To determine the total binding capacity of FMN to the heterodimer complex, ITC experiments were performed. ITC analysis showed that FMN binding to the FRedA/B heterodimer is an exergonic reaction with a ΔG = -13.93 kcal/mol and a K***_D_*** = 1.96 ± 0.13 μM. The reaction displays unfavorable entropy changes (TΔS = -2.67 kcal/mol) and favorable enthalpy changes (ΔH = -11.26 kcal/mol). Addition of FMN during ITC resulted in a 1.36:1 stoichiometric ratio of FMN:dimer (**Figure 4**). Given that these proteins purified with 0.59 moles of FMN per mole of heterodimer, the ITC results confirmed that the heterodimer contains two FMN binding sites. No FMN binding was observed with the individually purified FRedA and FRedB monomers. This result, along with size exclusion data, supports the obligate formation of the heterodimer complex prior to FMN binding.

### Characterization of the NADH Oxidase Activity of the *L. johnsonii* N6.2 FRedA/B Heterodimer

Individually, FRedA and FRedB were unable to oxidize NADH. Individually purified FRedA and FRedB were combined in a 1:1 ratio to determine their activity. Combined proteins showed a 75% lower NADH oxidase activity compared to co-purified FRedA/B (48.26 ± 6.6 μmol min^-1^ mg^-1^ vs. 209.74 ± 30.1 μmol min^-1^ mg^-1^, respectively). This assay suggested that the holoenzyme could be re-assembled and the activity could be recovered by mixing individually purified proteins in presence of FMN. This result also indicates that FMN is acquired after the protein is synthesized and it is not deeply buried in the core of the protein.

Determination of ROS during the enzymatic reaction indicated that the NADH oxidase activity correlated with the H_2_O_2_ accumulation. In light of these findings, the biochemical characterization was continued with copurified FRedA/B. Kinetic analysis indicated that the enzyme heterodimer followed Michaelis–Menten saturation using either FMN or FAD as electron acceptors, and NADH as an electron donor. Non-linear regression showed that the heterodimer has similar affinity for FMN and FAD (K_m_ = 20 and 19 μM respectively). However, the highest V_max_ and catalytic efficiency was observed with FMN over FAD (V_max_ = 203.55 ± 11.58 μmol min^-1^ mg^-1^ vs. 66.98 ± 4.1 μmol min^-1^ mg^-1^ and K_cat_ = 145.85 s^-1^ vs. 47.9 s^-1^, **Table 3**). Riboflavin could not be used as a substrate for FRedA/B, and β-NAD(P)H did not serve as an effective electron donor.

**Table 3:** Steady state kinetics of copurified FredA/B.

Substrate	V_max_ (μmol/min/mg)	K_m_ (μM)	k_cat_ (s^-1^)	k_cat_/K_m_ (s^-1^ M^-1^)
NADH	184.51 ± 8.4	59.68 ± 5.8	132.21	2.22E+06
FMN	203.55 ± 11.5	20.36 ± 2.6	145.85	7.16E+06
FAD	66.98 ± 4.1	19.85 ± 2.5	47.99	2.42E+06

FRedA/B was inactive at alkaline and neutral pH, with the highest NADH oxidase activity recorded at pH 5.5. Metal ion screening showed that the divalent cations Fe^2+^, Co^2+^, Cu^2+^, and Zn^2+^ significantly inhibited FRedA/B NADH oxidase activity. The characterized enzymes Yief (ChrR) from *E. coli* and FerB from *Paracoccus denitrificans* share 27% identity with FRedA, and 31 and 27% identity, respectively, with FRedB. Both of these proteins have been characterized for their ability to transfer electrons from NAD(P)H to multiple acceptors, including quinones (Ackerley et al., 2004a,b; Sedláček et al., 2014). Given their homology, we investigated the ability of the *L. johnsonii* FRedA/B to reduce quinones using a panel of substrates; yet no enzyme activity was detected.

FMN reductases are capable of both single and double electron transfer. To characterize the flow of electrons from NADH to O_2_ during FRedA/B activity, we followed the formation of H_2_O_2_ during catalysis. Concentrations of NADH and H_2_O_2_ were inversely correlated over the course of the reaction. We calculated the ratio of NADH oxidization rate to H_2_O_2_ production during the linear range of the reaction (V_0_). For each mole of NADH oxidized, 0.56 ± 0.01 moles of H_2_O_2_ were produced by FRedA/B. This result suggests that a single electron transfer to FMN forms a radical semiquinone intermediate with a simultaneous electron transfer directly to O_2_. Subsequently, the side product O_2_^•-^ suffers a spontaneous dismutation to the relatively more stable H_2_O_2_.

### LjPAS Affect the Formation Rate of the Active Complex

**FIGURE 5:**
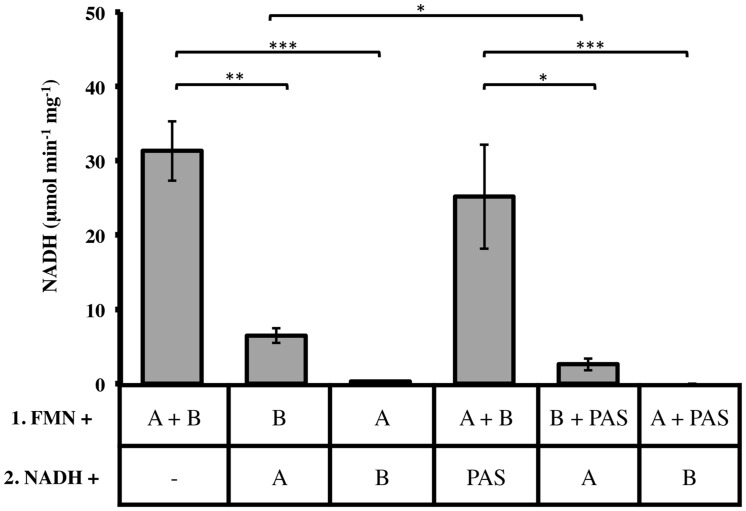
**Effect of monomer preincubation with FMN and LjPAS on heterodimer NADH oxidase activity.** Reaction mixtures contained: 40 μM FMN, 400 μM NADH, and 1 μg/mL each monomer in 100 mM MES buffer pH 5.5. Proteins were preincubated with FMN for 5 min as indicated in Row 1 before addition to the components included with NADH in Row 2. Reactions were continuously monitored at 340 nm. A = FRedA, B = FRedB, PAS = LjPAS. All reactions were performed in triplicate. Activities represent means ± standard deviations with ^∗^*p*-value < 0.05, ^∗∗^*p*-value < 0.01, and ^∗∗∗^*p*-value < 0.005.

Two important observations re-directed our research to investigate the relationships of LjPAS with FRedA and FRedB: (a) the fusion of LjPAS homologs to FRedA and FRedB homologs in related bacteria and (b) improvement of solubility of FRedA expressed in *E. coli* when LjPAS was coexpressed. Consequently, the effect of LjPAS on FMN reductase activity was investigated. To characterize the order of formation of the active heterodimer complex, individually purified monomeric units were preincubated with FMN before addition to the reaction mixture and the remaining monomeric unit. Preincubation of FRedB with FMN resulted in faster active complex formation compared to preincubation of FRedA with FMN (6.48 ± 1.22 μmol min^-1^ mg^-1^ and 0.27 ± 0.11 μmol min^-1^ mg^-1^). However, when monomers were combined and incubated with FMN together prior to addition of NADH, V_o_ was significantly higher (31.31 ± 3.94 μmol min^-1^ mg^-1^). These results suggest that FMN binding by FRedB monomer is a necessary first step to initiate active complex formation. Similar assays were carried out with the addition of purified LjPAS protein. The addition of LjPAS did not significantly slow the initial rate of the reaction after FRedA and FRedB were preincubated with FMN (25.16 ± 7.60 μmol min^-1^ mg^-1^). However, preincubation of FRedB with LjPAS significantly reduced V_0_ by 50% (6.8 ± 1.22 μmol min^-1^ mg^-1^ to 2.6 ± 0.81 μmol min^-1^ mg^-1^; **Figure 5**). Taken together these results suggest that LjPAS can affect the rate of catalysis either by affecting activity or, more likely, slowing down the interaction between the monomers. Once the heterodimer is formed in the presence of FMN, LjPAS has no anymore effects on NADH oxidase activity.

### FRedA and FredB Interact *In Vivo* in a Bacterial Two-Hybrid System

**FIGURE 6:**
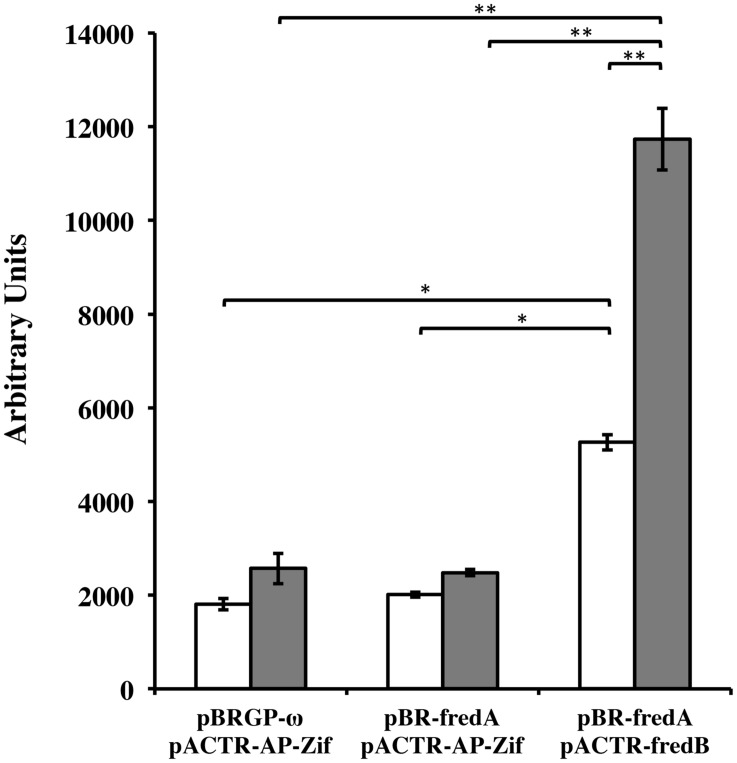
***Lactobacillus johnsonii* N6.2 FRedA and FRedB interact in a bacterial two-hybrid system.** Transcription activation stimulated by the interaction between FRedA and FRedB from *L. johnsonii* N6.2 was investigated using plasmid constructs pBR-FRedA and pACTR-FRedB in the *E. coli* LacZ reporter strain KDZifDΔZ. Cells were grown under static (white) or aerobic (gray) conditions, and AUs of β-galactosidase activity were determined following the hydrolysis of the CPRG substrate. All values were normalized for culture growth (OD_600_). All assays were performed in triplicate. β-galactosidase activities are reported as mean ± standard deviation with ^∗^*p*-value < 0.005 and ^∗∗^*p*-value < 0.001.

In order to confirm that FRedA and FRedB interact *in vivo*, a bacterial dual hybrid assay was performed. The plasmid pBRGP-ω was used to create the fusion of the *L. johnsonii* N6.2 FRedA gene to the ω subunit of the RNA Polymerase. Plasmid pACTR-AP-Zif was used to fuse the *L. johnsonii* N6.2 FredB gene to the zinc finger DNA binding protein of the murine Zif268 domain. Both plasmids were transformed into the reporter *E. coli* strain KDZifΔZ harboring the β-galactosidase reporter plasmid. Reporter strains containing a single FMN reductase reporter plasmid and one empty plasmid, as well as reciprocal clones, were constructed. The expression of the FRedA and FRedB reporter fusions strongly induced the transcription of β-galactosidase compared to control strains harboring a single protein (FRedA or FRedB) or empty plasmids (2,478 ± 54 AU vs. 11,733 ± 656 AU), supporting the *in vivo* interaction of these proteins in the creation of an active heterodimer complex *in vivo* (**Figure 6**). This system was also used to assess LjPAS interaction with the FRedA/B heterodimer. The two-hybrid assay carried out in the same *E. coli* strain with the addition of a third plasmid expressing LjPAS showed no differences in β-galactosidase activity.

### FRedA/B Relevant Structural Features

The predicted tertiary structure of individual FRedA and FRedB monomers indicated that the active protein is a member of the FMN reductase superfamily in the flavoprotein subgroup (COG0431). This is a large and heterogeneous group of proteins with undefined biological roles. Our search for proteins with solved crystal structures of high similarity identified a group of 20 homologs, based on the structural features predicted for each monomeric unit. The main scaffold for both monomers (FRedA or FRedB) was predicted based, primarily, on three crystallized proteins: *E. coli* quinone reductase ChrR (PDB ID: 3SVL), FMN reductase from *P. aeruginosa* (PDB ID: 1X77), and the NAD(P)H-dependent oxidoreductases from *P. denitrificans* (PDB ID: 3U7R). Overall, the proteins display the canonical α/β structure classic of the flavodoxin family. The central core is composed of five twisted parallel β-sheets flanked by a group of prominent alpha helices (three external and two internal). The side chains of the internal α3 and α4 helices, together with amino acids forming the shallow FMN binding pocket, comprise the main contacts of the active heterodimer unit.

**FIGURE 7:**
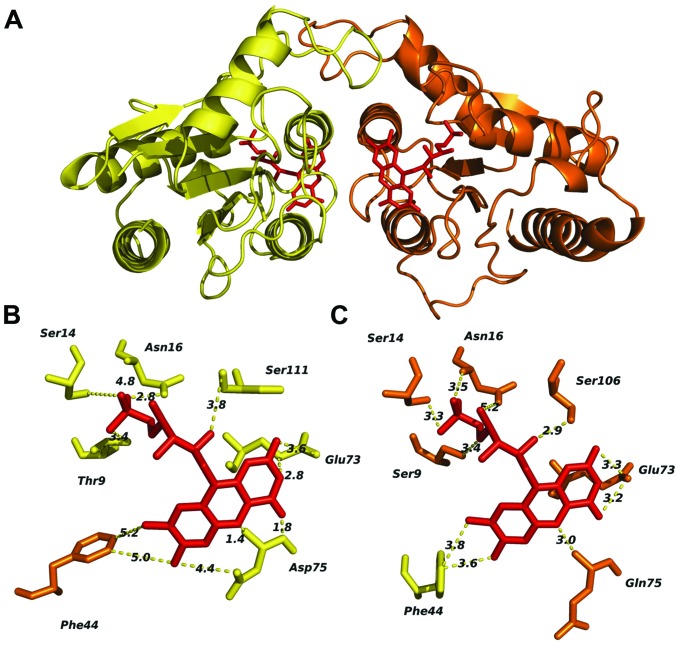
***In silico* analysis of the *L. johnsonii* FRedA/FRedB heterodimer complex and identification of amino acids involved in FMN pocket formation.** The FRedA/B heterodimer was modeled based on the structurally homologous chromate reductase YieF from *E. coli.* **(A)** Predicted structure of the heterodimer with FRedA (yellow) and FRed B (orange). **(B)** Predicted FRedA FMN binding pocket. **(C)** Predicted FRedB FMN binding pocket with relevant amino acids.

The two FMN binding pockets of FRedA/B, as well as those described in closely related flavodoxin homo-oligomers, are formed on opposite sides near the dimer interface. Crystallized FRedA and FRedB homologs form active homodimers and homotetramers, resulting in two or four identical FMN binding pockets per active complex. As a result of the formation of the heterodimer, each active FRedA/B complex contains two asymmetric FMN binding pockets near the dimer interface (**Figure 7A**). Each pocket is formed by a projecting loop that follows the α2 helix, and two short loops connecting the central β sheets with the α3 and α4 helices. The presence of these loops as structural parts of the active pocket suggests a high level of flexibility in flavin cofactor binding. The α3 and α4 helices of the second unit form the “wall” of the pocket, which helps to hold the isoalloxazine ring nearly parallel to the main axis of protein symmetry. As a consequence, the cofactor is completely exposed to the solvent. As well as in the homologs, fully conserved Glu73, together with Asp75 (pocket A) and Gln75 (pocket B), are critical to stabilize the isoalloxazine ring of FMN (**Figures 7B,C**). A flexible, but fully conserved non-polar Phe44, from the adjacent monomer in each case, protrudes toward FMN and helps to shape each cavity. The ribityl phosphate group rests on the surface of the main axis of the groove with the phosphate portion deeply buried within the cavity and interacting with Ser14 in both catalytic pockets. The hydroxyl groups of Thr9 in pocket A and Ser9 in pocket B, together with a fully conserved Ser (106 and 111 respectively) also contribute to FMN binding. Consequently, the redox center of the molecule is positioned flat toward the entrance of the cavity. Our enzymological studies suggest that the attachment of FRedB to FMN could be the first step toward the formation of the active complex. These results are in agreement with the higher conservation of amino acids between characterized homologs such as FerB and the predicted FMN pocket formation in FRedB. Intriguingly, none of these structural features help to explain the lack of FRedA/B activity toward the quinone substrates evaluated in this work.

### H_2_O_2_ Production by *L. johnsonii* FRedA/B Increases Epithelial Cell ROS

**FIGURE 8:**
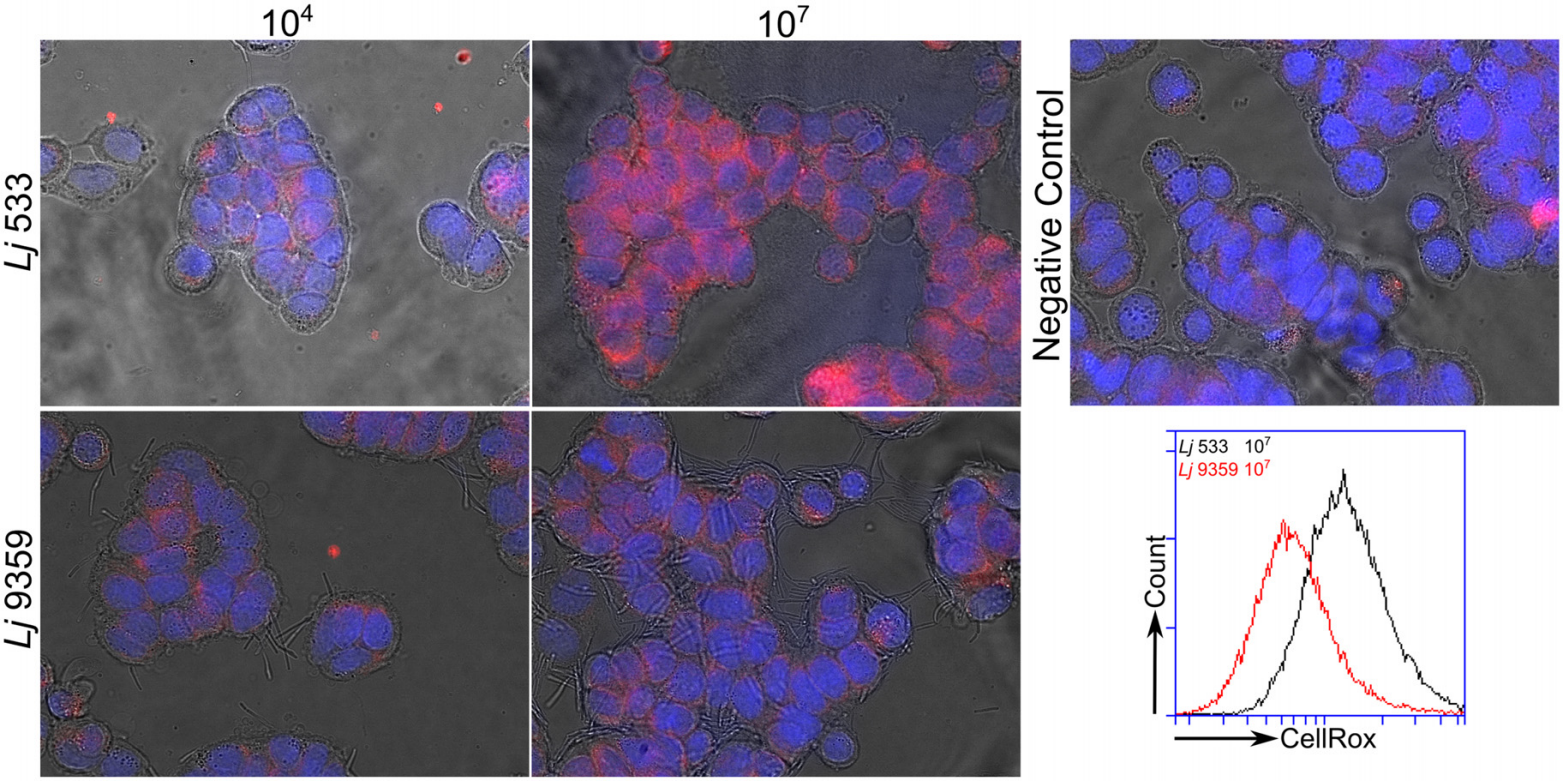
**Fluorescence microscopy images of ROS produced by *L. johnsonii*.** Hydrogen peroxide production by the wild type *L. johnsonii* NCC 533 strain (Lj533) and the isogenic double FMN reductase knock out mutant NCC 9359 (Lj9359) were compared using CellRox Deep red dye and fluorescence confocal microscopy in HT-29 human intestinal epithelial cell cultures. This assay demonstrates that during epithelial cell co-culture, the *L. johnsonii* FMN reductase heterodimer contributes significantly to H_2_O_2_ production. HT-29 cells were treated with *L. johnsonii* at 10^4^ and 10^7^ colony forming units/mL. A control image shows epithelial cells without *L. johnsonii* treatment.

Several groups have recently shown that commensal bacteria can increase host cell H_2_O_2_ production by stimulation of innate signaling pathways and NADPH oxidase activity (Swanson et al., 2011; Valladares et al., 2013). This increase in intracellular ROS production has been shown to have significant beneficial effects on cell migration and inflammatory signaling (Lin et al., 2009; Swanson et al., 2011). The role of bacterially sourced ROS in microbe–host interactions has not been thoroughly investigated. After characterizing H_2_O_2_ production by the FRedA/B heterodimer, we sought to evaluate the role of this protein complex and its H_2_O_2_ product in *L. johnsonii*-intestinal epithelial tissue culture. We co-cultured human HT-29 intestinal epithelial cells with our wild type *L. johnsonii* N6.2 isolate in the absence and presence of catalase. We also evaluated the role of H_2_O_2_ production in the commercial probiotic *L. johnsonii* NCC533 and the H_2_O_2_-negative FMN-reductase knockout derivative of NCC533, NCC 9359 (Hertzberger et al., 2014). We quantified the intracellular ROS levels using the ROS-specific dye CellRox Deep Red (Life Technologies, Grand Island, NY, USA) coupled with fluorescence microscopy and flow cytometry. Intestinal epithelial cells incubated with *L. johnsonii* N6.2 or *L. johnsonii* NCC533 stained significantly higher for ROS than cells incubated with these strains plus catalase or the FMN reductase heterodimer knockout *L. johnsonii* NCC9359 (**Figure 8**). These results show that the H_2_O_2_ production by the FMN reductase heterodimer of *L. johnsonii* largely contributes to epithelial cell ROS levels in our tissue culture assay.

## Discussion

Although H_2_O_2_ production by commensal lactobacilli was first described over 60 years ago (Wheater et al., 1952), the proteins responsible for elevated ROS production in commensal acidophilus complex *Lactobacillus* spp. and the role of these ROS in *Lactobacillus*-host interactions *in vivo* is still poorly understood. On this front, we recently described the H_2_O_2_-mediated modulation of the key immunoregulatory enzyme indoleamine 2,3-dioxygenase in host intestinal epithelial cells with *L. johnsonii* N6.2. As a direct consequence of such interaction the tryptophan/kynurenine ratios were systemically modified in BBDP rats used as experimental host (Valladares et al., 2013).

Our goal when initiating this study was to identify and characterize new pathways in *L. johnsonii* N6.2 responsible for H_2_O_2_ production. Several groups have suggested that central metabolic enzymes such as pyruvate oxidase and lactate oxidase are primary contributors to ROS production in acidophilus-complex lactobacilli. The commercial probiotic strain *L. johnsonii* NCC533, shares high genome sequence identity with the *L. johnsonii* N6.2 isolate. A work in NCC533 published by Hertzberger et al. (2013) showed that inactivation of the gene encoding pyruvate oxidase in *L. johnsonii* NCC533 did not influence H_2_O_2_ production by this closely related strain. As part of our investigation directed to identify new mechanisms of ROS producing in *L. johnsonii* N6.2, we performed RNA-seq to assess the transcript level response toward high and low oxygen environments. The analysis of the transcriptome profile obtained indicated that, alongside the differential regulation of several pathways extensively described before, a new miss-annotated gene was identified. The product of this gene is a small soluble protein (30 Kd) carrying a PAS folded domain as unique and relevant characteristic. Three significant observations that T285_08005 could encode a non-yet described regulatory protein: (1) it is differentially expressed in response to different oxygen tension; (2) it was not possible to predict or associate the encoded protein to any enzymatic activity; (3) the presence of a characteristic PAS sensor domain in the predicted protein structure. The resulting identification of LjPAS as a protein of interest, and the subsequent bioinformatic analysis of PAS-FMN reductase fusion proteins in related Firmicutes, directed our identification, cloning and purification of two sequentially encoded FMN reductases in *L. johnsonii* N6.2. During our analysis of these proteins, Hertzberger et al. (2014) published a follow-up study identifying two genes, *lj0548* and *lj0549*, encoding FMN reductases responsible for the elevated H_2_O_2_ phenotype of *L. johnsonii* NCC533. The corresponding protein products in *L. johnsonii* NCC533 are identical to FRedA and FRedB from *L. johnsonii* N6.2. Although their work confirmed the role of these genes in H_2_O_2_ production in this bacterium, the proteins were only partially purified and the genetic regulation was no studied. Here, we report the cloning, purification to homogeneity, and the characterization of this heterodimeric FMN reductase from *L. johnsonii* N6.2. In addition, we evaluate the relationship of this heterodimer with a widely encoded PAS sensor domain protein. Finally, we show that this heterodimeric FMN reductase is central in *L. johnsonii* H_2_O_2_ production and consequently to bacteria and host redox-based interactions. We found that FRedA/B synthesis is not regulated at transcriptional level but post-translationally, by interacting with LjPAS.

All previously reported bacterial FMN reductases form homo-oligomers with flavin catalytic centers, and utilize NADH and/or NAD(P)H as electron donors. In agreement with the evaluation of the reductase activity in cell lysate fractions described by Hertzberger et al. (2014), we found that FRedA/B formed an active heterodimer *in vitro.* In contrast, we found that riboflavin did not serve as an effective flavin cofactor and the purified FRedA/B use only NADH as electron donor. Here, we show unequivocally that FRedA and FRedB interact *in vivo* through the use of a bacterial two-hybrid system, as well as *in vitro* using affinity purified proteins and size exclusion chromatography. We observed that the heterodimer formation is necessary for flavin cofactor binding. Although FMN reductases form a large group of promiscuous enzymes capable of reducing a variety of compounds, quinone reduction is hypothesized to be their chief physiological role (Eswaramoorthy et al., 2012; Sedláček et al., 2014). Our analysis of FRedA/B from *L. johnsonii* N6.2 demonstrated that this heterodimer performs a single electron transfer, forming a FMN semiquinone intermediate with an O_2_^-^ as reaction by product. Although an obligate two-electron transfer has been described for homologous flavoproteins, the FMN reductase FerB from *P. denitrificans* was also shown to perform single electron transfers from NADH to naphthoquinones (Sedlácek et al., 2009). Furthermore, FRedA/B was not capable of using menaquinone or menadione as enzymatic substrates. This lack of activity correlated with the absence of an endogenous quinone biosynthetic pathway in *L. johnsonii* and related lactobacilli.

We hypothesize that the enzymatic complex herein studied may constitute a distinctive system for coping with oxygen in a bacterium lacking the canonical antioxidant molecules and enzymes such as glutathione, superoxide dismutase, and catalase. The rapid consumption of free molecular oxygen by FRedA/B may also serve to prevent more deleterious chemistry elsewhere inside the cells or complete inhibition of highly oxygen-sensitive reactions. This system will work synergistically with others like the enhanced uptake of manganese to minimize the formation of the highly reactive iron centers. With a decreased presence of iron clusters, H_2_O_2_ produced intracellularly could more easily diffuse out of the cell to be catabolized by other microbes or host antioxidant enzymes (Culotta and Daly, 2013). In this way, *L. johnsonii* can minimize Fenton like reactions improving its ability to cope with intercellular oxidative stress.

The role of LjPAS is sustained by two important evidences collected in our assays. In first instance the repression of the gene expression (more than 3 fold) in cells grown under low oxygen concentration. In addition to that, once purified, LjPAS has the ability to slow down FRedA/B reductase activity rate *in vitro*. Considering that FRedA and FRedB genes are constitutively expressed under oxygen rich and oxygen starved environments; we hypothesize that LjPAS will help the cells better adapt their metabolism during eventual transitions from aerobic to anaerobic conditions. On this scenario our model will depend of protein–protein interactions. Indeed, the slowdown of the active complex formation *in vitro* is an indirect evidence of such interaction. This fact is reinforced by the improvement of protein recovery once LjPAS is co-expressed with each FMN monomer. Although, the *E. coli* two-hybrid assays expressing the *L. johnsonii* proteins confirmed the interaction of FRedA/B *in vivo*, this system was not appropriate for evaluating the physical interaction of LjPAS with the heterodimer. Several reasons may explain the failure of the *E. coli* two hybrid systems to evaluate LjPAS interactions with the complex. We hypothesize that the protein–protein interactions could be weak or the contact could not be stable enough to measure differences. Also, the intracellular *E. coli* redox context could be substantially different to that observed in *L. johnsonii* hampering interactions. In addition; the PAS folded domain, a potential sensor center, could be affected by the binding of unknown small ligands affecting the interaction.

The formation of a heterodimeric FMN reductase is novel as well as the involvement of a small protein controlling its activity by direct interaction. A follow up work will be necessary to elucidate the molecular aspects of the heterodimer formation in the native biological context and shed light on LjPAS biological value. Our future efforts will be directed to identify the redox signaling molecules involved in regulating the complex formation. Further studies will also be necessary to fully characterize the physiological function and regulation of this protein complex in *L. johnsonii* and its protein fusion homologs in other Firmicutes.

## Conflict of Interest Statement

The authors declare that the research was conducted in the absence of any commercial or financial relationships that could be construed as a potential conflict of interest.

## Abbreviations

AAP: 4-aminoantipyrine
AUs: arbitrary units
BLAST: basic local alignment search tool
CRPG: chlorophenol red-β-D-galactopyranoside
EtOH: ethanol
H_2_O_2_: hydrogen peroxide
His_6_: hexahistidine tag
HPLC: high performance liquid chromatography
IPTG: isopropyl β-D-1-thiogalactopyranoside
ITC: isothermal titration calorimetry
MES: 2-(*N*-morpholino)ethanesulfonic acid
O_2_^•-^: superoxide
PAGE: polyacrylamide gel electrophoresis
PAS: Per-Arnt-Sim
PCR: polymerase chain reaction
ROS: reactive oxygen species
SDS: sodium dodecyl sulfate
STRING: Search Tool for the Retrieval of Interacting Genes/Proteins
TRIS: tris(hydroxymethyl)aminomethane
