# Supplementary material for: H2O2 production rate in Lactobacillus johnsonii is modulated via the interplay of a heterodimeric flavin oxidoreductase with a soluble 28 Kd PAS domain containing protein
Source: Front Microbiol. 2015 Jul 14;6:716. doi: 10.3389/fmicb.2015.00716 (PMC4500961; doi:10.3389/fmicb.2015.00716)
Supplement: Supplementary file 1 [file Table_1.PDF]

Supplementary Table 1: RNA-Seq. loci corresponding to reads with significant changes (> 3 fold) in aerated cultures.

Locus Tag	Putative Gene Product	RPKM Aerated	RPKM Static	Fold Change (Aerated/Static)
T285_07615	Bi-functional Alcohol/Acetylaldhyde dehydrogenase	253.72	3045.57	-12.00
T285_07995	Pheromone response surface protein PrgC	31.56	256.07	-8.11
T285_00345	Hypothetical protein	172.91	1146.53	-6.63
T285_07590	Hypothetical protein	345.38	1850.05	-5.36
T285_08935	Cytochrome d ubiquinol oxidase subunit I	184.06	906.11	-4.92
T285_01070	Hypothetical protein	6.71	28.76	-4.29
T285_01075	PTS system, cellobiose-specific IIB component	64.65	271.98	-4.21
T285_08940	Cytochrome d ubiquinol oxidase subunit II	164.08	642.27	-3.91
T285_01880	Cell envelope-associated transcriptional attenuator LytR-CpsA-Psr	17.80	69.26	-3.89
T285_00525	Methylated-DNA--protein-cysteine methyltransferase	62.92	241.73	-3.84
T285_07990	ABC transporter ATPase component	4.75	17.36	-3.66
T285_07070	PTS system, fructose- and mannose-inducible IIA component	22.78	80.63	-3.54
T285_07065	PTS system, fructose- and mannose-inducible IIB component	31.71	111.80	-3.53
T285_08530	Beta-lactamase class A	41.77	145.06	-3.47
T285_07060	PTS system, fructose- and mannose-inducible IIC component	28.79	99.55	-3.46
T285_02235	Response regulator of the LytR/AlgR family	78.49	255.98	-3.26
T285_08950	Transport ATP-binding protein CydC	57.51	184.71	-3.21
T285_08945	Transport ATP-binding protein CydD	105.47	337.65	-3.20
T285_08005	Fumarate reductase, flavoprotein subunit precursor (LjPAS)	122.48	375.45	-3.07
T285_00840	Phosphoenolpyruvate carboxykinase	14.94	45.02	-3.01
T285_00785	Ribonucleotide reductase of class III (anaerobic), activating protein	5182.77	1725.74	3.00
T285_03405	Hypothetical protein	666.56	213.98	3.12
T285_08040	PEP-dependent sugar phosphotransferase EIIAB, probable mannose specific	20.69	6.23	3.32
T285_06805	Hypothetical protein	4.30	1.29	3.33
T285_04690	Hypothetical protein	1.07	0.32	3.33
T285_08030	PTS system, mannose/fructose/sorbose family, IIC component	22.13	6.58	3.36
T285_07445	Oxidoreductase, Gfo/Idh/MocA family	293.14	82.59	3.55
T285_04355	Hypothetical protein	23.71	6.58	3.61
T285_03450	Aminopeptidase C	245.24	66.94	3.66
T285_00275	Adhesin	3524.01	801.66	4.40
T285_00285	Cell surface protein precursor	795.23	160.76	4.95
T285_08010	L-cystine uptake protein TcyP	404.18	71.88	5.62
T285_05040	Hypothetical protein	69.79	11.87	5.88
T285_05045	Methyltransferase	828.42	119.80	6.92
T285_05085	Iron-sulfur cluster assembly ATPase protein SufC	1719.95	241.39	7.13
T285_05080	Iron-sulfur cluster assembly protein SufD	1679.23	209.83	8.00
T285_05075	Cysteine desulfurase, SufS subfamily	1205.86	135.02	8.93
T285_03430	Oligopeptide ABC transporte	1989.08	200.94	9.90
T285_05065	Putative iron-sulfur cluster assembly scaffold protein for SUF system	1446.27	144.56	10.00
T285_05060	Iron-sulfur cluster assembly protein SufB	673.15	65.52	10.27
T285_05055	PaaD-like protein involved in Fe-S cluster assembly	1198.48	110.00	10.90
T285_03395	Glucosamine--fructose-6-phosphate aminotransferase	1151.39	102.90	11.19
T285_05050	Manganese transport protein MntH	737.49	54.46	13.54
