# Supplementary material for: H2O2 production rate in Lactobacillus johnsonii is modulated via the interplay of a heterodimeric flavin oxidoreductase with a soluble 28 Kd PAS domain containing protein
Source: Front Microbiol. 2015 Jul 14;6:716. doi: 10.3389/fmicb.2015.00716 (PMC4500961; doi:10.3389/fmicb.2015.00716)
Supplement: Supplementary file 2 [file Table_2.PDF]

Supplementary Table 2: Validation of RNA-Seq. transcript level changes by RT-PCR

Locus Tag	Putative Gene Product	Change in gene expression measured by:	
		RNASeq	qRT-PCR
T285_07615	Bi-functional alcohol/acetylaldehyde dehydrogenase	-12.00	-10.93 ± 0.53
T285_08935	Cytochrome d oxidase 1	-4.92	-5.11 ± 1.16
T285_08940	Cytochrome d oxidase 2	-3.91	-4.06 ± 0.32
T285_07060	Fructose/Mannose Inducible IIC Component	-3.46	-4.36 ± 0.02
T285_08005	Fumarate reductase, flavoprotein subunit precursor (LjPAS)	-3.07	-1.65 ± 0.09
T285_00265	Lactate Dehydrogenase	-2.93	-1.86 ± 0.24
T285_08070	Surface protein Rib	-2.28	-2.58 ± 0.43
T285_06810	Glutamine synthetase type I	1.93	1.87 ± 0.07
T285_08030	Mannose/Fructose/Sorbose Family IIC Component	3.36	4.1 ± 1.05
T285_07445	Oxidoreductase MocA family	3.55	3.32 ± 0.32
T285_00275	Hypothetical Protein - Gram + Anchor Domain	4.40	4.02 ± 0.53
T285_05060	Iron-Sulfur Cluster Protein SufB	10.27	9.16 ± 1.56
T285_05050	Manganese Transporter MntH	13.54	15.7 ± 3.07
